# Skin Diseases and Syphilis

**Published:** 1893-12

**Authors:** 


### SKIN DISEASES AND SYPHILIS.

EDITED BY DR. M. B. HUTCHINS.

### A REMARKABLE CASE OF HERPES ZOSTER UNIVERSALIS.

Dr. Pio Colombini, Assistant in the Dermatological Clinic of the University of Siena. (*Commentario Clinico delle Malattie Cutanee e Genito-Urinarie*, Nos. 1, 2, 3, 4, Siena, 1893.)

Dr. Colombini describes at length a very rare manifestation of Herpes Zoster. It was universal, and "mapped out" the cutaneous nerve distribution of the whole of the body. Two excellent colored lithographs, executed at the deaf-mute institution at Siena, showing the anterior and posterior aspects of the body in the erect position, reveal at a glance the nature of the affection. As far as is known only three cases have hitherto been recorded where Herpes Zoster has implicated the whole of the integument: I. De Amici's case, reported in the *International Journal of Medical Sciences*, Fourth Year, 1882, p. 72; II. Pennetti's case, following influenza, *Riforma Medica*, Sixth Year, 1890, Vol. I., p. 738; III. Pugliesi's case, *Riforma Medica*, Seventh Year, 1891, Vol. II., p. 689.

Dr. Colombini's case is that of a young man, ætat. thirty, a peasant by birth and occupation. Parents healthy, as also the other members of the family. When twenty-seven he had malarial fever, which lasted some months, leaving him very weak; he had no skin trouble or any neuralgic affections. He had another attack of malaria, the present one, when he was thirty, and this time suffered from intense neuralgia, and from a burning sensation all over the body. Within a week of the onset of the malaria his skin was affected with universal herpes zoster, and it was at this time that Dr. Colombini saw him. The neuralgia was very severe, he was feverish, and the skin presented in the various nerve cutaneous areæ patches of erythema with congeries of vesicles in various stages of progress—in short, the typical eruption of zoster. The conjunctivæ, nasal, oral and anal mucosæ also showed vesicles. The rash was bilaterally symmetrical, the right half of the body being worse than the left. There was everywhere a diminution of common sensation in the erythematous areæ, and local discomfort due to the implication of the orificial mucosæ.

There was a large amount of albumen present in the urine during the first few days. It gradually disappeared as the rash improved. The spleen was distinctly enlarged, and the febrile paroxysms were typically malarial in character. The treatment was, internally the administration of quinine, locally boric lotions, boric collutoria, boric collyria. During convalescence arsenic was given to combat the persistent neuralgiæ in the various regions, especially in the trifacial nerve area. He was under treatment for six weeks. The contents of those vesicles which had become purulent revealed staphylococcus pyogenes aureus in predominating numbers. Besides pus corpuscles, Colombini was able to satisfy himself that certain Marchiastellate bodies were present, clearly recalling to mind similar forms described by Fava and Celli as constantly present in the blood during paludal paroxysms. According to these observers these forms represent the second or reproductive stage of the plasmodium malariæ.

Dr. Colombini proceeds to discuss the diagnosis; but a glance at the two illustrations clearly show that the affection could not be mistaken for either eczema universalis or pemphigus acutus (with millet-like blebs). Finally he comes to the question of etiology; this he goes into thoroughly, and on the strength of this case more especially, Colombini concludes:

1. Zona or zoster is a general, febrile, spontaneous, acute and almost cyclic (*i. e.*, running a definite course) disorder, ending always in recovery and conferring immunity. It is a general affection with a circumscribed local manifestation.

2. The characteristic feature of zoster is rather the specific neuropathy that produces the cutaneous eruption than the eruption itself. It is not enough simply to refer zoster to trophic disturbance; on the contrary it must be regarded as a specific neuropathy.

Dr. Colombini distinctly refers his remarkable case to the consequences of malaria in an overwrought subject.—*British Journal of Dermatology*, October, 1893.

### COSMETICS.

Dr. Robt. B. Morison, of Baltimore, read a paper upon this subject at the seventeenth annual meeting of the American Dermatological Association.

For the removal of freckles he advises the following:

℞. Corrosive sublimate, grs. 7.
Destilled water f℥ 6.
Spirits camphor, f℥ ½.
Rose water, ℥ 5.

M. This is applied upon three or four thicknesses of linen, cut to fit, at night—removed when dry. Redness occurs after a few nights application, epidermis then peeling in fine scales. Following ointment then to be used :

℞. Cetacii,
Cerae alb., āā 7.
Ol. amygdalæ, 14.
Præcip. alb., 1.40.
Ac. salicyl., 1.

M. Use night and morning, gently rubbed in for five minutes with a *clean* finger.

He says the above is satisfactory treatment, and that the return of the freckles can usually be prevented.

[Removing the full thickness of the epidermis will remove freckles, but the prevention of their return should be promised with caution.—ED.]

An idea for the ladies is the use of the galvanic current to produce a reddening of the cheeks. The current was carefully applied to the cheeks and produced a reddening that lasted several hours.

---

### CAMPHORATED CARBOLIC ACID.

Dr. D. E. English of Milburn, N. J., contributes an article upon this mixture to the September 30th *N. Y. Medical Record*.

It is made by putting together in a bottle one part by weight of liquefied carbolic acid and three parts by weight of gum camphor. In a short time a smooth, transparent, oily liquid is formed, changing with age from colorless to pink. "It mixes freely with olive oil and vaselene, and dissolves iodoform in proportion of one to twelve, completely disguising the odor of iodoform." "Dissolves freely in alcohol but will not mix with water or glycerine." Causes no more pain than a 1–60 solution of carbolic acid.

He thinks he has aborted boils and cured carbuncles by injecting it around the lesion, as carbolic acid is sometimes used. With carron oil 1–20 used successfully in burns of first and second degrees.

[A similar formula makes a valuable anti-pruritic agent, but it may be found that it *does* produce more irritation than a 1–60 carbolic solution, if used pure.—ED.]

---

### OIL OF WINTERGREEN IN ALOPECIA.

Dr. Hallopeau, of Paris (*Bulletin Gen. de Thérapeut.*, May 8, 1893), reported to the Société de Thérapeutique the case of a young man whom he had treated with great success with this remedy, combined with three parts of ether. For experimental purposes it was only applied to one side of the head, the essence of canella—a substance in good repute for the treatment of area—being used for the other side. After five months of treatment the difference between the two sides was very marked, that treated with oil of wintergreen (Oleum Gualtheriæ) being almost healthy, while the other showed only downy hairs. The remedy has an agreeable odor, and provokes neither pain nor redness of the skin, in favorable contrast to most parasiticides, which are powerful irritants.—*British Journal of Dermatology*, October, 1893.

---

### OINTMENT FOR PIGMENTATION OF PREGNANCY.

(*Union Médicale*, November, 1892.)

℞. Zinci oxid. pur., gr. iv.

Hydrarg. oxid. flav., gr. xvi.

Ol. ricini,

Theobromatis olei, āā ℨ iijss.

Otto ros., gutt. x.

Sig.: The ointment for use twice daily—some to be allowed to remain on at night.—*British Journal of Dermatology*, October, 1893.

